# Increase of isoflavones in the aglycone form in soybeans by targeted crossings of cultivated breeding material

**DOI:** 10.1038/s41598-019-46817-1

**Published:** 2019-07-17

**Authors:** Jegor Miladinović, Vuk Đorđević, Svetlana Balešević-Tubić, Kristina Petrović, Marina Ćeran, Jelena Cvejić, Mira Bursać, Dragana Miladinović

**Affiliations:** 1 Soybean Department, Institute of Field and Vegetable Crops, 21000 Novi Sad, Serbia; 2 Department of Pharmacy, Faculty of Medicine, University of Novi Sad, 21000 Novi Sad, Serbia; 3 Industrial Crops Department, Institute of Field and Vegetable Crops, 21000 Novi Sad, Serbia

**Keywords:** Agricultural genetics, Plant breeding

## Abstract

Isoflavones are a group of phytoestrogens, naturally-occurring substances important for their role in human health. Legumes, particularly soybeans (*Glycine max* (L.) Merr.), are the richest source of isoflavones in human diet. Since there is not much current data on genetics of isoflavones in soybean, particularly in the aglycone form, elucidation of the mode of inheritance is necessary in order to design an efficient breeding strategy for the development of high-isoflavone soybean genotypes. Based on the isoflavone content in 23 samples of soybeans from four different maturity groups (00, 0, I and II), three crosses were made in order to determine the inheritance pattern and increase the content of total isoflavones and their aglycone form. Genotype with the lowest total isoflavone content (NS-L-146) was crossed with the low- (NS Zenit), medium (NS Maximus), and high- (NS Virtus) isoflavone genotypes. There were no significant differences in the total isoflavone content (TIF) between F_2_ populations, and there was no transgression among genotypes within the populations. Each genotype within all three populations had a higher TIF value than the lower parent (NS-L-146), while genotypes with a higher TIF value than the better parent were found only in the NS-L-146 × NS Zenit cross. However, significant differences in the aglycone ratio (ratio of aglycone to glycone form of isoflavones) were found between the populations. The highest aglycone ratio was found in the NS-L-146 × NS Maximus cross. The results indicate that the genetic improvement for the trait is possible.

## Introduction

Isoflavones are naturally occurring group of phytoestrogens. They are mostly found among the members of the *Fabaceae* family, primarily soybeans and red clover^1^. Soybean (*Glycine max* (L.) Merr.) is an industrial crop with a stable production growth. Soybean is the fourth major crop in the world, following cereal crops, such as maize, wheat and rice, which are the main source of human food. Since soybeans and soy products are the richest source of isoflavones in human diet^2^, the content of isoflavones in new soybean cultivars has become a trait with increased significance. Regarding differences in bioavailability of individual isoflavones, it is important to establish the form in which they are present in the seed^3^. Soybeans contain twelve different phytoestrogens. Daidzein, genistein and glycitein are the aglycones which can form three glucoside forms, a β-glucoside, a 6″-O-malonyl-glucoside and a 6″-O-acetyl-glucoside^4^. The isoflavone aglycones are absorbed faster and in greater amounts than their glucosides in humans^5^.

Considering the increasing presence of soybean in human diet, the nutritive value of soybean and soy products have become the focus of numerous studies in agronomy, production technology, and medicine. Moreover, the key role of diet in the prevention of some serious pathologies, such as cardiovascular diseases, atherosclerosis and cancer, has been globally recognized. Soybeans have been extensively studied around the world due to their presence in human diet and many health benefits which have been attributed to the consumption of soy and soy products. Special attention has been placed on the research on soybean effects on certain types of cancer. Studies regarding the relationship between soy intake and breast cancer^6–11^ showed that increased amounts of soybean in daily diet reduce the risk of breast cancer in Asian women, which is partly attributed to the presence of isoflavones. In addition, it has been confirmed that isoflavones, primarily their aglycones (genistein and daidzein), is involved in a number of biological activities including breast and prostate cancer chemo-preventive activity, and the ability to modify carcinogenesis, e.g. initiation, promotion, and cancer progression^12–15^.

Increasing the content of isoflavones in new varieties of soybean, particularly in their aglycone form, is one of the most important objectives in modern soybean breeding programs. The influence of various factors on isoflavones content and composition in soybeans has been studied by different authors. Content and composition of isoflavones in soybeans largely depend on the soybean genotype. While the origin of soybeans does not have a major impact on these characteristics^16,17^, the maturity group i.e. the length of vegetation period does^18,19^. According to previous studies, the amount and the composition of isoflavones in soybeans are affected by the environment, primarily by the temperature during the vegetation period and storage^20–23^.

Previous investigations of the inheritance of isoflavone content in soybeans indicate that genetic improvement for these traits should explore the additive genetic variance in superior lines, or the cytoplasmic effect and the epistatic interactions between cytoplasmic and nuclear genes to obtain the maximum gain in selection^24^. Our previous results showed that F_1_ soybean progenies could increase isoflavone content^25^, and that the content and composition of isoflavones could be passed from the parental genotypes to the hybrids, and therefore utilized for breeding soybean cultivars with desirable traits^26^.

Since there is not much data on genetics of isoflavones in soybean, elucidation of the mode of inheritance is necessary in order to design an efficient breeding strategy for development of high-isoflavone soybean genotypes. The aim of this study was to investigate the inheritance of total isoflavones and their aglycone form in F_2_ generations, as well as prospective increase of the total isoflavone content in genotypes with the low isoflavone content.

## Material and Methods

### Crosses

Based on the isoflavone content in 23 different soybean genotypes from four different maturity groups (00, 0, I, and II), three crosses were made in order to determine the inheritance pattern and increase the content of total isoflavones and their aglycone form. The entire list of isoflavone content in these 23 soybean genotypes can be found as Supplementary Table S1. Genotype with the lowest total isoflavone content (NS-L-146) was crossed with the low- (NS Zenit), mid- (NS Maximus), and high- (NS Virtus) isoflavone content genotypes. Offspring of these crosses was grown the following year as the F_1_ generation at the same locality, according to the standard methodology^27^. The same stands for the F_2_ generation.

### Isoflavone analysis

Seeds used for *in vitro* experiments were collected at full maturity. The collected seeds were then extracted by the method of Andlauer, Martena, and Furst^28^. Powdered soybean seeds (500 mg) were defatted by hexane extraction (2 × 10 ml, 30 min, and subsequent centrifugation, 30 min, 1780 rcf) and then extracted for 2 h with 8 ml methanol/water (4:1, v/v) and centrifuged (30 min, 1780 rcf). Prior to HPLC injection, each extract was filtered using Agilent technologies Teflon filters (0.45 μm, Delaware, Wilmington).

Individual isoflavones were identified and quantified according to the method of Lee *et al*.^4^, with minor modifications. Separation was achieved on 5 μm Zorbax SB C18 HPLC column (150 × 4.6 mm), with Zorbax SB C18 guard column. Mobile phase consisted of two solvents. Solvent A was 1% (v/v) acetic acid in water and solvent B was 100% acetonitrile. Analysis was conducted under the following conditions: 0–5 min 85% A; 5–44 min from 85 to 65% A; 44–45 min from 65 to 85% A, and 45–50 min 85% A. The column temperature was 25 °C, a solvent flow rate 0.6 ml/min, and injection volume 10 μl. The spectra were collected between 240 and 400 nm by DAD, and components in the eluate were detected at 270 nm.

**Figure 1:**
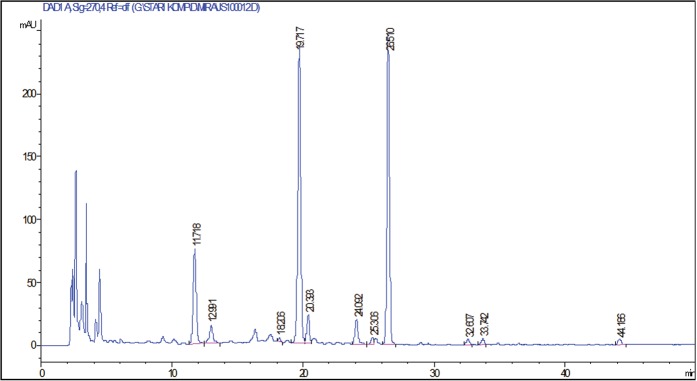
Chromatogram of NS-Virtus soybean sample. *Retention time (min) of detected compound: 11.7 – daidzin (DZI), 12.9- glycitin (GLYI), 18.2- genistin (GEI), 19.7 - malonyl daidzin (MDZI), 20.3 – malonyl glycitin (MGLYI), 24.1 – acetyl daidzin (ACDZI), 25.3 – acetyl glycitin (ACGLYI), 26.5 – malonyl genistin (MGEI), 28.9 – acetyl genistin (ACGEI), 32.6 – daidzein (DZ), 33.7 – glycitein (GLY), 44.2 – genistein (GE).

**Figure 2:**
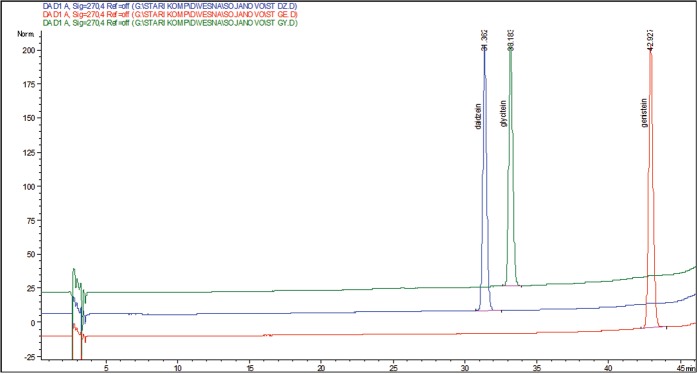
Chromatogram of aglycone standard compounds. *Retention time (min) of standard compound: 31.3 – daidzein (DZ), 33.2 – glycitein (GLY), 42.9 – genistein (GE).

Isoflavones were identified by comparing the retention times in HPLC chromatograms and UV spectral patterns with those of standard compounds and literature data^4,28^ (Figs 1 and 2).

Isoflavone concentrations were quantified by external standard (five-point regression curves, r ≥ 0.9997) of daidzein, glycitein and genistein standard compounds. Standard solutions were made by dissolving standard compounds in mixture of methanol/water (4:1, v/v) and linearity was studied for each compound in the range of 0.5 to 50 mg/l (0.5 0.1 2.0 3.0, 5.0, 10.0, 25.0 and 50.0). As only standard phytoestrogen aglycones were used, the content of the corresponding glycoside forms was obtained by calculation. For this purpose, calibration curves of the corresponding aglycone compounds were used and corrections for differences in molecular weight between aglycones and glucosides were applied following the pattern given by Romani *et al*.^29^:

$$c\,(glucoside)=c\,(corr\,aglycone)\cdot \frac{Mr\,(glucoside)}{Mr\,(corr\,aglycone)}$$

### Statistical analysis

All sample measurements were done in triplicate. Data were processed by analysis of variance (ANOVA) using statistical program package STATISTICA v. 12.0^30^. Statistical comparisons between samples were performed by Student’s t-test for independent analysis. Differences were considered significant at p < 0.05. The method proposed by Mahmud and Kramer^31^ was used to estimate broad-sense heritability on a single-plant basis. The method involves the measurement of a character on F2 plants of a single-cross population and on the inbred parents used to form the population. The formula for estimating heritability presented by these investigators is

$${h}^{2}=\frac{{\sigma }_{F2}^{2}-\sqrt{({\sigma }_{P1}^{2})({\sigma }_{P2}^{2})}}{{\sigma }_{F2}^{2}}$$

Where:

*h*^2^ = heritability, ${\sigma }_{F2}^{2}$ = variance of F_2_, ${\sigma }_{P1}^{2}$ = variance of P_1_, ${\sigma }_{P2}^{2}$ = variance of P_2_

Principle Component Analysis was done in statistical program SPSS 10.0.

## Results

Broad-sense heritability for analyzed traits was medium to low. It ranged from 0.20 for acetyl genistin of F_2_ NS-L-146 × Virtus, to 0.61 for acetyl glycitin of the same cross (Table 1).

**Table 1:** Broad-sense heritability of isoflavone content.

F2 population	DZ	DZI	ACDZI	MDZI	GLY	GLYI	ACGLYI	MGLYI	GE	GEI	ACGEI	MGEI	Aglycone	TIF	TDZI	TGLY	TGEI
NS-L-146 × Virtus	0.36	0.43	0.39	0.41	0.33	0.52	0.61	0.51	0.48	0.52	0.20	0.41	0.31	0.35	0.46	0.39	0.48
NS-L-146 × Zenit	1^*^	0.38	0.58	0.42	1^*^	0.31	1^*^	0.54	1^*^	0.52	1^*^	0.41	0.21	0.48	0.44	0.42	0.41
NS-L-146 × Maximus	1^*^	0.44	0.51	0.18	0.39	0.44	0.48	0.49	0.47	0.31	1^*^	0.38	0.3	0.39	0.38	0.41	0.38

^*^Calculated value, parental values are 0 and progeny values are different from zero.

DZ – diadzein, DZI – daidzin, ACDZI - acetyl daidzin, MDZI - malonyl daidzin, GLY-glycitein, GLYI – glycitin, ACGLYI- acetyl glycitin, MGLYI - malonyl glycitin, GE-genistein, GEI – genistin, ACGEI - acetyl genistin, MGEI - malonyl genistin, TIF- total isoflavones, TDZ- total daidzein, TGLY- total glycitein, TGE – total genistein.

**Figure 3:**
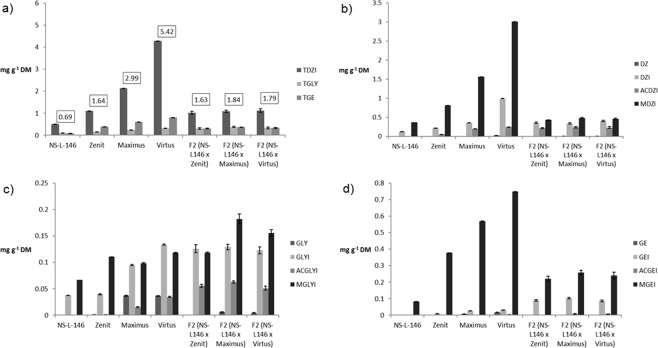
Isoflavone content and composition of parents and F2 populations. (**a**) Total isoflavone content (TDZ- total daidzein, TGLY-total glycitein, TGE – total genistein). (**b**) Diadzein and and diadzein conjugates content (DZ –diadzein, DZI – daidzin, ACDZI - acetyl daidzin, MDZI - malonyl daidzin). **(c**) Glycitein and glycitein conjugates content (GLY-glycitein, GLYI – glycitin, ACGLYI- acetyl glycitin, MGLYI - malonyl glycitin). **(d**) Genistein and genistein conjugates content (GE-genistein, GEI – genistin, ACGEI - acetyl genistin, MGEI - malonyl genistin).

Three F_2_ populations, obtained from the crosses between the low isoflavone genotype and three other parents (low, medium, high), had the different mode of inheritance of isoflavone content (Fig. 3a).

Dominant isoflavone in soybean seed is diadzein, followed by genistein and glycitein. Significant differences in the content of individual isoflavones were not found between populations, except for the content of total glycitein. Malonyl daidzin, followed by daidzein, were the dominant forms of diadzein (Fig. 3b). Content of acetyl daidzin in the F_2_ population made from NS-L146 × Zenit was higher compared to the both parents. The average content of malonyl daidzin in populations F_2_ (NS-L146 × Maximus) and F_2_ (NS-L146 × Virtus) low and similar to NS-L-146. Glycitin and malonyl glycitin were the dominant forms of glycitein (Fig. 3c). All three populations had a similar, unusually high content of acetyl glycitin, regardless of the parents used for crosses. Furthermore, there were several lines in populations F_2_ (NS-L146 × Maximus) and F_2_ (NS-L146 × Zenit) with glycitein different from zero. Malonyl glycitin content in populations F_2_ (NS-L146 × Maximus) and (NS-L146 × Virtus) exceeded the mid-parent value. In parental genotypes, malonyl genistin was the dominant form of genistein while in F_2_ populations genistein was found in the form of malonyl genistin and genistin (Fig. 3d). There were no significant differences between F_2_ populations regarding their genistein content. Overall, F_2_ populations had a similar content of diadzein and genistein (glycon and aglycon form) and population average value was closer to the lower parent. On the other hand, dominance or overdominance of the better parent was observed regarding glycitein content in F_2_ populations.

**Figure 4:**
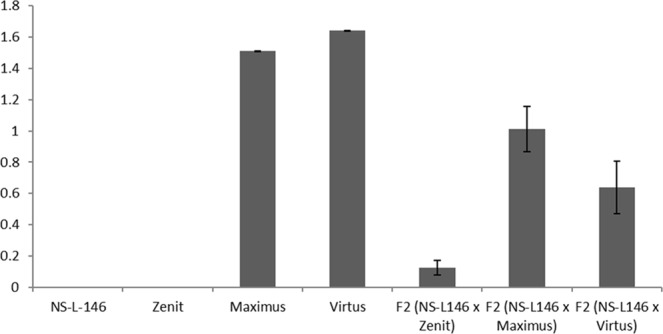
Aglycone ratio in parental genotypes and the coresponding F_2_ populations.

Ratio of aglycone and glycone forms of isoflavones is an important indicator of isoflavone content, due to the faster absorption of the aglycone form in the gastrointestinal tract. Population average indicates that the aglycone ratio showed the mid-parent value mode of inheritance. Even from crosses between parents with zero value aglycone ratio, some progenies had a certain amount of aglycone form of particular isoflavones (Fig. 4).

**Figure 5:**
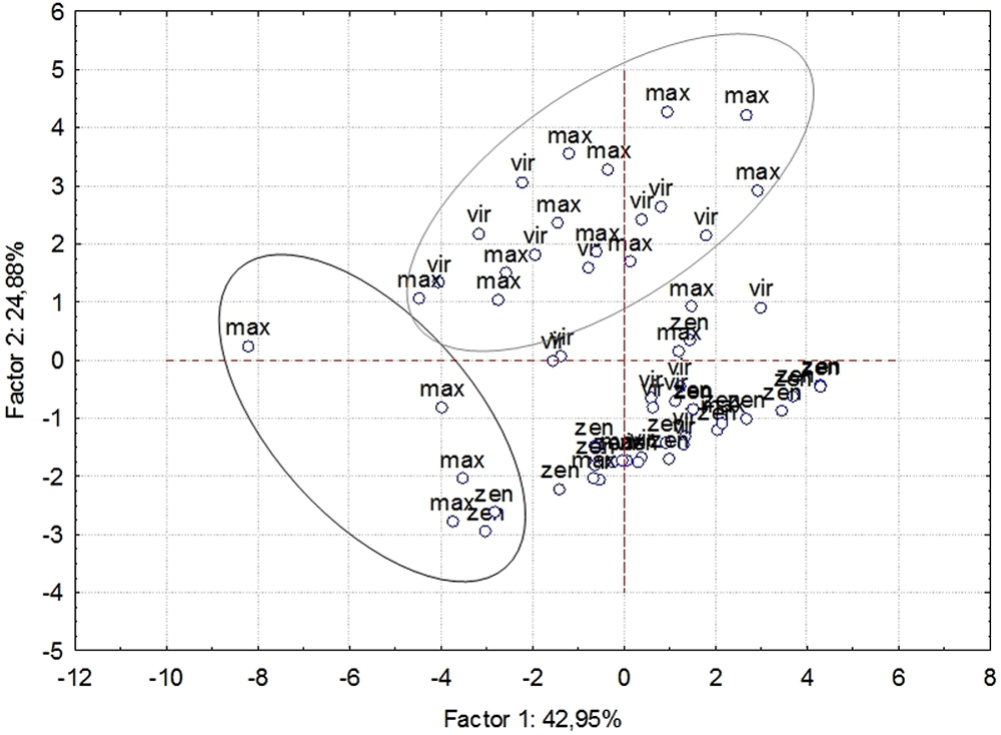
Principal Component Analysis of isoflavone content in F_2_ populations. Black circle – high total isoflavone lines, gray circle – high aglycone ratio (Zen - F2 (NS-L146 × Zenit), Max - F_2_ (NS-L146 × Maximus), Vir - F2 (NS-L-146 × Virtus)).

The first two principal components of F_2_ lines isoflavone content, explained 68% of the total trait variation from the initial dataset (Fig. 5), 43% for principal component I and 25% for principal component II. The first principal component was mostly related to acetyl, 7-glycoside form of diadzein, malonyl glycitin, genistin, and total isoflavones, while the second principal component explained diadzein, glycitein, acetyl genistin content and the aglycone ratio. Although average isoflavone content showed little difference between populations, the principal component analysis divided F_2_ lines into two groups. The first group consisted of lines with high isoflavone content (Fig. 5, red circle), while the second group consisted of lines with higher aglycone ratio. On the other hand, the lines obtained from the NS-L146 × Zenit cross were grouped on the PCA plot, while the lines from the other two crosses were more disperse, which indicates low breeding potential of the NS-L146 × Zenit cross.

## Discussion

The prospect of increasing total isoflavones in genotypes with low isoflavone content was tested by choosing four parents, based on the tests of isoflavone content in 23 different soybean genotypes.

Mean values of total isoflavone content were the same for all three F_2_ populations, regardless of the parents used in the cross. Crossing the parents which had low total isoflavone content (NS-L-146 by Zenit) resulted in the inheritance of total isoflavone content from the better parent (Zenit), which leads to the conclusion that the inheritance is associated with the better parent. However, the same level of total isoflavones was obtained in crosses between the lines with low total isoflavone content and cultivars with medium and high total isoflavone content, such as Maximus and Virtus. Therefore, it was not possible to make the final decision on the mode of inheritance, as this trait is affected by many genes with few individual effects and it is under strong influence of the environmental factors^32,33^.

The content of diadzein and diadzein conjugates, as well as genistein and genistein conjugates, followed the same pattern of inheritance as total isoflavones. At the same time, they were the dominant forms of isoflavones in all the analyzed samples. This is in agreement with the previous observations, stating that soybeans and soy foods usually contain similar amounts of genistein and daidzein and a much lower amount of glycitein^34^. On the other hand, glycitein and glycitein conjugates had the highest variation, which is in agreement with the result of Gutierrez-Gonzales *et al*.^35^. The content of glycitein conjugates in F_2_ populations is especially interesting. There was no difference in glycitin content between the three populations and the parent with the highest glycitin content (Virtus). Acetyl glycitin content of the three F_2_ populations was equally higher, compared to the parent with the highest acetyl glycitin content (Virtus), which is interesting given the fact that F_2_ (NS-L-146 × Zenit) derived from the parents with no detected acetyl glycitin content. Low isoflavone levels of the offspring indicate that maternal effect has a significant role in isoflavone inheritance, as showed by Chiari *et al*.^24^.

Aglycone ratio of the F_2_ populations derived from the cross between NS-L-146, Maximus and Virtus, approximately had an intermediate type of inheritance. However, the cross between NS-L-146 and Zenit, which did not contain free isoflavone forms, resulted in some F_2_ lines with a certain amount of free isoflavone forms. Inheritance of aglycone ratio in soybeans is scarcely documented, but our results point out the aglycone ratio as one of the prospective breeding goals in future soybean breeding programs.

The use of Principal Component Analysis to estimate isoflavone content in individual lines provides a clearer view of the variation of this trait in F_2_ populations. Most lines from the F_2_ (NS-L-146 × Zenit) were grouped on the PC graph, indicating their low variation and thus low significance in breeding for this trait. On the other hand, besides their total isoflavone content, the position of several lines from F_2_ (NS-L-146 × Maximus) on the graph confirms high variability for this trait, so they will be used for further breeding programs. Lines from F_2_ (NS-L-146 × Virtus) and F_2_ (NS-L-146 × Maximus), have a higher aglycone ratio than the better parent. Therefore, they will serve as important starting material in the development of genotypes with increased content of isoflavones in the aglycone form, which is an important objective in modern soybean breeding programs.

According to the obtained results, future soybean breeding for changed isoflavone content and composition is possible. Breeding success is important considering the constant need for improvement i.e. increase of isoflavone content in cultivars with the desired agronomic traits, and the rising importance of isoflavones in human diet. In their choice of parental components, breeding programs should aim at obtaining the highest possible variability, which would be possible by using both the average and high isoflavone genotypes. Further research should focus on detecting the mode of inheritance in order to achieve major breakthrough in breeding for this significant trait.

## Supplementary information

Dataset 1
