# Author Correction: Increase of isoflavones in the aglycone form in soybeans by targeted crossings of cultivated breeding material

**DOI:** 10.1038/s41598-019-53778-y

**Published:** 2019-11-15

**Authors:** Jegor Miladinović, Vuk Đorđević, Svetlana Balešević-Tubić, Kristina Petrović, Marina Ćeran, Jelena Cvejić, Mira Bursać, Dragana Miladinović

**Affiliations:** 1 Soybean Department, Institute of Field and Vegetable Crops, 21000 Novi Sad, Serbia; 2 Department of Pharmacy, Faculty of Medicine, University of Novi Sad, 21000 Novi Sad, Serbia; 3 Industrial Crops Department, Institute of Field and Vegetable Crops, 21000 Novi Sad, Serbia

Correction to: *Scientific Reports* 10.1038/s41598-019-46817-1, published online 17 July 2019

The original version of this Article contained an error in Affiliation 2, which was incorrectly given as ‘Department of Pharmacy, Faculty of Medicine, 21000, Novi Sad, Serbia’. The correct affiliation is listed below:

Department of Pharmacy, Faculty of Medicine, University of Novi Sad, 21000, Novi Sad, Serbia

This has now been corrected in the HTML and PDF versions of this Article.

